# How is acceptance of the brain disease model of addiction related to Australians’ attitudes towards addicted individuals and treatments for addiction?

**DOI:** 10.1186/s12888-014-0373-x

**Published:** 2014-12-24

**Authors:** Carla Meurk, Adrian Carter, Brad Partridge, Jayne Lucke, Wayne Hall

**Affiliations:** The University of Queensland, UQ Centre for Clinical Research, Royal Brisbane and Women’s Hospital Site, Herston, Queensland 4029 Australia

**Keywords:** Public attitudes, Brain disease model of addiction, Neuroethics, Alcohol, Heroin, Stigma

## Abstract

**Background:**

We investigated whether beliefs about addiction being a ‘disease’ or ‘brain disease’, and holding certain beliefs about addiction aetiology, are associated with public views about addicted persons and support for different types of treatment, coerced treatment and punishment for addiction.

**Methods:**

Data were collected as part of the 2012 Queensland Social Survey, a computer assisted telephone interview of 1263 residents of Queensland, Australia. Participants were presented with scenarios of two addicted males, one who was addicted to heroin and the other addicted to alcohol. Participants were then asked a series of questions for both characters.

**Results:**

There was widespread support for all treatment modalities (alcohol: 80.8-98.0%, heroin: 89.9-97.2%). There was less support for coerced treatment for alcohol than heroin addiction (alcohol: 41%, heroin: 71%, χ^2^ = 273.90, p < 0.001). Being 35 years of age or older (alcohol: OR = 0.58 (0.37-0.91), heroin: OR = 0.49 (0.28-0.85)) and having 15 or more years of education (alcohol: OR = 0.60 (0.44-0.81), heroin: 0.55 (0.40-0.75)) predicted less support for coerced treatment. 31.7% of respondents agreed heroin use should be punished by imprisonment and being 35 years of age or older (OR = 0.51 (0.33-0.80)) predicted lack of support. The sample agreed that an alcohol or heroin dependent person would suffer career damage (alcohol: 96.2%, heroin: 98.9%), marriage breakdown (alcohol: 92.2%, heroin: 97.3%) and get in trouble with the law (alcohol: 92.3%, heroin: 98.9%). Respondents expressed more comfort with encountering alcohol rather than heroin addicted persons in the workplace or at a dinner party. Beliefs that addiction was a ‘brain disease’ or a ‘disease’ did not predict any of these attitudes. Beliefs about addiction aetiology were inconsistent predictors of outcomes measured.

**Conclusions:**

Age and educational attainment were the most consistent predictors of stigmatising beliefs and beliefs about coercion and punishment. Beliefs that addiction is a ‘disease’ or a ‘brain disease’ were not associated with an overall reduction in beliefs about stigma, coercion or punishment. Beliefs in different causes of addiction were not consistent predictors of beliefs about stigma, coercion or punishment.

## Background

Neuroscience research on the effects of chronic addictive drug use on brain function has been used to argue that addiction is a chronic relapsing ‘brain disease’ [1,2]. In particular, the brain disease model holds that the brain changes associated with repeated drug use impair autonomy and restrict addicted persons’ ability to freely refrain from using drugs [3].

The implications of this model for how addicted individuals are viewed and treated are hotly contested. Advocates argue that acceptance of the brain disease view will reduce the stigmatisation that many people with an addiction experience by showing that an inability to refrain from using drugs arises not from personal qualities such as moral ‘weakness’ but as a result of drugs ‘hijacking’ the brain’s reward system [4]. The hope is that reducing stigmatisation and self-blame will reduce social barriers to treatment and increase treatment seeking. Proponents also argue that elucidation of the neural mechanisms underlying addiction will lead to more effective ways of treating addiction, including psychopharmacological and perhaps neurosurgical approaches. An optimistic view of the brain disease model suggests that it provides a rationale for offering treatment to drug addicted offenders rather than incarcerating them for crimes related to their addiction (e.g. drug possession, crimes committed in order to obtain drugs) [4].

Critics of the brain disease model of addiction argue that it will fail to deliver these benefits or perhaps produce more negative consequences for addicted persons. Some argue that placing responsibility for addiction on the individual’s ‘brain’ neglects the role of the social environment – ranging from family to workplace, to broader structural and cultural dynamics – as a contributor to addiction. They argue that the brain disease model individualises responsibility and may increase funding for health programs that focus on ‘medical’ aspects of addiction at the expense of social ones [5]. Some detractors of the brain disease view suggest that it could absolve addicted persons of responsibility for drug use (i.e. ‘it’s not me, it’s my brain’), increase their fatalism (i.e. ‘my brain’s damaged, there’s nothing I can do’) [6], or encourage people to start using drugs if they believe that it will be easy to quit with medical assistance [5].

There is also some evidence that believing that an addicted person’s brain is damaged or ‘diseased’ may, in fact, *increase* stigmatisation [7]. Moreover, the brain disease model could be used as justification for coerced treatment on the grounds that addicted persons are not fully autonomous in making decisions about drug use. This may leave addicted persons vulnerable to the coercive use of extreme, and potentially dangerous, therapies such as ablative neurosurgery [8,9] or deep brain stimulation [10].

The distinction between a ‘brain disease’ model of addiction and the more general ‘disease’ concept reflects important historical as well as scientific influences on the framing of addiction. While the ‘brain disease’ concept is of relatively recent origin, the ‘disease’ concept has a longer history of use through its association with, and the prominence of, Alcoholics Anonymous (AA) [11,12]. Within the AA framing, alcoholism as a ‘disease’ is not associated with a specific biological aetiology. Rather, it is viewed as a disease in the general sense of being an incurable individual affliction that can only be managed through complete abstinence from alcohol [11].

Our previous qualitative research with members of the general public suggests that (neuro)biological understandings often co-exist with other beliefs about addiction. Beliefs that addiction is a ‘disease’ or a ‘brain disease’ imply biological conceptions of addiction to the exclusion of other causes of addiction [13-16]. Thus, support for a (neuro)biological role in addiction is not synonymous with accepting the brain disease model. This study examines the extent to which predictions made by proponents and critics of the brain disease model are reflected in public attitudes towards addicted persons, policy and treatments for addiction - part of our broader anticipatory analysis of the public health and policy implications of addiction neuroscience research [6]. Here, we report findings from a sample drawn from the Australian population that examined how labelling addiction as a ‘brain disease’ was related to the public’s views of addicted persons, the best treatments for addiction, and whether coercion or punishment of addicted persons was a justifiable approach. We compare the social consequences of a ‘brain disease’ label with the older ‘disease’ conception. We also examined whether beliefs about the social and biological causes of addiction were associated with particular views about how addiction should be treated. We discuss our findings in terms of their implications for communicating messages about addiction.

## Methods

### Sampling and recruitment

Participants were interviewed as part of the 2012 Queensland Social Survey (QSS), a computer-assisted telephone interview of 1263 residents in the State of Queensland, Australia. QSS is administered by the Population Research Laboratory at Central Queensland University Australia and the inclusion of our questions was approved by the Human Ethics Research Review Panel at that University. Participants provided verbal consent to participate. Households were randomly selected using a database of randomly generated telephone landline numbers and sampled to achieve an equal gender quota.

The survey had a response rate of 35.3%. In comparison to the Australian Bureau of Statistics 2011 census data for Queensland, participants over the age of 55 were over-represented and those under the age 35 were under-represented (Table 1) [17,18]. The under-representation of younger persons could partly reflect shifts among this demographic towards cellular phones rather than landlines [19]. Descriptive statistics were estimated to have a sampling error of plus or minus 2.7%, at a 95% confidence level.

**Table 1:** **Demographic characteristics of survey participants** [13]

Gender	Male			Female		
	49.7%			50.3%		
Age	18-24	25-34	35-44	45-54	55-64	65+
	4.3%	7%	16.5%	18.6%	22.6%	30.6%
Years of education	1-10 years	11-12 years		13-14 years		15+ years
	24.9%	21.5%		11.3%		41.6%

### The survey instrument

QSS is a large omnibus survey that includes questions from multiple research bodies and organisations on a range of topics. We included questions that explored beliefs about: the causes of addiction; its treatment; the role of coercion and punishment; and stigma, discrimination and dangerousness.

Participants answered these questions after being asked to reflect on two hypothetical scenarios describing addicted men: i) John who was addicted to alcohol; and ii) Peter who was addicted to heroin. We chose these two drugs in order to contrast attitudes towards a common legal drug (alcohol) and a well-recognised illegal drug (heroin).

Participants rated their agreement on a five-point scale (strongly agree, agree, disagree, strongly disagree, do not know) to each of seven possible causes of each type of addiction, namely i) bad character; ii) addictive personality; iii) psychological problems; iv) chemistry in the brain; v) the way he was raised; vi) stress; and vii) a genetic or inherited problem.

Participants were asked about the extent of their agreement with the propositions that each type of addiction is i) a disease and ii) a brain disease.

Familiarity with addiction was assessed by asking participants whether they, or someone they knew personally, had ever had a problem with i) alcohol or ii) heroin. The response options were i) no; ii) yes, I have; iii) yes, someone close to me has; and iv) yes, both myself and someone close to me has [13].

Participants rated their beliefs about the value of different treatments for addiction on a four-point scale (very helpful, somewhat helpful, not helpful, don’t know/unsure). Questions asked were:

John/Peter should …

i) talk to a doctor or psychiatrist about his addiction
ii) take medication prescribed by a doctor to help him quit
iii) visit a mental health worker, such as a counsellor, social worker, or psychologist
iv) get help from close family friends or a support group like alcoholics/narcotics anonymous

They were also asked to rate their agreement on questions about coerced treatment for heroin and alcohol, and imprisonment for heroin addiction, on a five-point scale (strongly agree, agree, disagree, strongly disagree, don’t know/unsure) as follows:

i) John/Peter should be forced into treatment for his alcohol/heroin addiction
ii) Peter should go to prison for his illicit drug use

Stigma, discrimination and dangerousness were measured using a variation of the Attitudes to Mental Illness Questionnaire (AMIQ) [20], a validated five question instrument for assessing stigmatising attitudes towards those with mental illness and addiction. Participants rated their views on a five-point scale of agreement (as above) or likelihood (very likely, quite likely, unlikely, very unlikely, don’t know/unsure) to the following questions:

i) John’s/Peter’s career will be damaged by his alcohol/heroin addiction
ii) I would be comfortable if John/Peter was my colleague at work
iii) I would be comfortable inviting John/Peter to a dinner party
iv) How likely do you think it would be for John’s/Peter’s wife to leave him?
v) How likely do you think it would be for John/Peter to get in trouble with the law?

### Data analysis

Five logistic regressions were conducted to investigate factors associated with beliefs about coerced treatment (alcohol and heroin), the degree to which participants felt ‘comfortable’ with addicted persons (alcohol and heroin) and whether they supported imprisonment for addiction (heroin only). AMIQ score for each respondent was calculated by adding together values for each of the five questions, scored on a scale [−2,2] and calculating the mean score [−10,10] [20]. Positive numbers denote a positive disposition towards a person or group and negative numbers denote a negative disposition. Data were analysed using SPSS v.20 [21].

### Variables

Following exploratory data analysis, the demographic variables included as predictors of attitudes in the logistic regressions we conducted were: gender (male = 0, female = 1); age (18–34 years = 0, age 35+ years = 1); and education (1–12 years = 0, 13–14 years = 1, 15+ years = 2). The binary variable ‘familiarity’ was based on answers to questions about personal experience with addiction. Those who had or knew someone who had had a problem with alcohol/heroin use were classed as having familiarity with addiction (no = 0, yes = 1). 62% of respondents had themselves experienced or had someone close to them experience problematic alcohol use. 19% of respondents reported having familiarity with problematic heroin use. In both cases, it was mostly someone close to the participant rather than the participant who had experienced problematic substance use (54% for alcohol and 15% for heroin).

As described previously [13], we created three variables (Table 2) that measured beliefs about addiction aetiology (disagree = −1, neutral/don’t know = 0, agree = 1). Our decision to group variables was based on our previous qualitative investigations as well as the thematic categories identified in the literature [15,16,22,23]:

**Table 2:** **Beliefs about causes of and labels for addiction**

	**Alcohol**			**Heroin**		
	**Agree**	**Disagree**	**Neutral/Don’t know**	**Agree**	**Disagree**	**Neutral/Don’t know**
Biological	48.1%	23.5%	28.3%	34.8%	31.7%	33.4%
Social-Environment	49.4%	16.5%	34.0%	33.0%	27.5%	39.5%
Character	59.6%	29.5%	10.9%	62.1%	24.9%	13.1%
Addiction is a brain disease	33.8%	40.9%	24.9%	32.8%	43.9%	22.7%
Addiction is a disease	66.6%	24%	9.3%	53.2%	35.7%	10.6%

1. ‘Biological’: indicating participants’ agreement that addiction is caused by i) chemistry in the brain; or is ii) genetic/inherited;
2. ‘Socio-environmental’: including i) the way he was raised; or ii) stress;
3. ‘Personal qualities’: including i) psychological problems; ii) addictive personality; or iii) ‘bad character’.

Responses to the four and five-point scales for agreement, likelihood and helpfulness were re-coded into binary variables (negative = 0, affirmative = 1) as follows: disagree/strongly disagree = negative = 0, agree/strongly agree = affirmative = 1; unlikely/very unlikely = negative = 0, very likely/quite likely = affirmative = 1; not helpful = negative = 0, very helpful/somewhat helpful = affirmative = 1.

Don’t know/unsure responses to these questions (ranging from 1.9%-13.8% of total response for each question) were discarded from analysis. Overall high rates of agreement relating to treatment made logistic regression analysis infeasible for these questions. The two questions from the AMIQ pertaining to ‘comfort’ were combined, by adding the dichotomized variables together, to create a comfort scale (not comfortable at either a dinner party and/or in the workplace = 0, comfortable at either a dinner party or in the workplace =1).

## Results

### Beliefs about treatment

There was strong support for all treatment modalities for both the heroin and alcohol addicted characters (Table 3). Participants were most likely to agree that a person should speak to family, friends, or attend a support group like Alcoholics Anonymous or Narcotics Anonymous (98% for alcohol, 97.2% for heroin). There was somewhat less, but still high levels of, support for using prescribed medication (80.8% for alcohol, 89.8% for heroin).

**Table 3:** **Participants’ beliefs about the treatments an addicted person should seek**

	**Alcohol**		**Heroin**	
**Peter/John should …**	**Affirmative (%)**	**Negative (%)**	**Affirmative (%)**	**Negative (%)**
Get help from close family friends or a support group like alcoholics/narcotics anonymous	98.0	2.0	97.2	2.8
Talk to a doctor or psychiatrist about his addiction	95.3	4.7	96.5	3.5
Visit a mental health worker, such as a counsellor, social worker, or psychologist	92.0	8.0	94.7	5.3
Take medication prescribed by a doctor to help him quit	80.8	19.2	89.8	10.2

### Beliefs about coerced treatment of addiction

There was significantly greater support for forcing Peter into treatment for his heroin addiction (71%) than for forcing John into treatment for his alcohol addiction (41%) (χ^2^ = 273.90, p < 0.001). Beliefs about whether addiction was a disease or a brain disease were not associated with support for coerced treatment (Table 4). There was only one significant association between beliefs about how addiction was caused and beliefs about coerced treatment for either alcohol or heroin: those who agreed that alcohol addiction was caused by personal qualities were 1.71 times more likely to agree that John should be forced into treatment than those who disagreed (OR = 1.71, 1.24-2.38).

**Table 4:** **Beliefs about whether an addicted person should be coerced into treatment for addiction**

**Peter/John should be forced into treatment**		**Alcohol addiction**		**Heroin addiction**	
		**OR**	**95% CI**	**OR**	**95% CI**
**Age**	**18-34**	1.00		1.00	
	**≥35**	0.58*	0.37-0.91	0.49*	0.28-0.85
**Gender**	**Male**	1.00		1.00	
	**Female**	1.24	0.93-1.65	0.73*	0.54-0.99
**Years of education**	**1-12**	1.00		1.00	
	**13-14**	0.75	0.46-1.22	0.76	0.46-1.25
	**15+**	0.60**	0.44-0.81	0.55***	0.40-0.75
**Familiarity with addiction**	**No**	1.00		1.00	
	**Yes**	0.72*	0.54-0.97	0.69	0.47-1.01
**Disease**	**No**	1.00		1.00	
	**Yes**	1.04	0.73-1.50	1.13	0.75-1.70
**Brain disease**	**No**	1.00		1.00	
	**Yes**	1.17	0.82-1.65	1.10	0.71-1.71
**Biological causes**	**Disagree**	1.00		1.00	
	**Neutral/DK**	1.07	0.72-1.59	1.03	0.68-1.55
	**Agree**	1.05	0.71-1.55	0.74	0.47-1.16
**Social-environmental causes**	**Disagree**	1.00		1.00	
	**Neutral/DK**	0.76	0.50-1.14	1.04	0.71-1.51
	**Agree**	1.12	0.75-1.66	0.91	0.61-1.36
**Personal qualities**	**Disagree**	1.00		1.00	
	**Neutral/DK**	1.39	0.80-2.42	1.26	0.69-2.30
	**Agree**	1.71**	1.24-2.38	1.34	0.93-1.92

*p < 0.05, **p < 0.01, ***p < 0.001, OR odds ratio, CI confidence interval, DK don’t know.

There was less support for coerced treatment of both alcohol and heroin addiction among older and more highly educated individuals. In the case of alcohol addiction, those 35 years of age and older were 0.58 times as likely as those aged 18–34 (OR = 0.58, 0.37-0.91) to agree that John should be forced into treatment. In the case of heroin addiction, those 35 years of age and older were 0.49 times as likely as those aged 18–34 (OR = 0.49, 0.28-0.85) to agree that Peter should be forced into treatment. Having 15 or more years of education was associated with lower support for coerced treatment in the case of both alcohol (OR = 0.60, 0.44-0.81) and heroin addiction (OR = 0.55, 0.40-0.75) compared to those with 1–12 years of education.

People who had experienced their own, or another’s alcohol addiction were 0.72 times less likely than those with no familiarity with alcohol addiction to agree that John should be coerced into treatment (OR = 0.72, 0.54-0.97). No association was evident between familiarity with heroin addiction and views on coerced treatment. Females were 0.73 times less likely than males to agree that Peter should be forced into treatment for his heroin addiction (OR = 0.54-0.99). We observed no association between gender and beliefs about coerced alcohol addiction treatment.

### Beliefs about imprisonment of addicted individuals

Approximately one-third (31.7%) of the sample agreed that Peter should go to prison for his heroin addiction. Table 5 shows that people 35 years of age or older were approximately half as likely as those aged 18–34 to agree that he should be imprisoned (OR = 0.51, 0.33-0.80). People who were neutral or did not know whether addiction had biological causes were one-third less likely than those who disagreed that heroin addiction had biological causes to think that Peter should go to prison (OR = 0.61, 0.41-0.91). Agreement that addiction had biological causes was not significantly associated with support for imprisonment. People who agreed that heroin addiction was caused by personal qualities were 2.3 times more likely to agree that Peter should go to prison than those who disagreed (OR = 2.30, 1.57-3.35). No other factors were significant but belief that addiction was a ‘disease’ was of borderline significance in predicting lower support for imprisonment (OR = 0.68, 0.46-1.00, p = 0.05).

**Table 5:** **Beliefs about imprisonment for heroin addiction**

**Peter should go to prison for his illicit drug use**			
		**OR**	**95% CI**
**Age**	**18-34**	1.00	
	**≥35**	0.51**	0.33-0.80
**Gender**	**Male**	1.00	
	**Female**	0.98	0.73-1.33
**Years of education**	**1-12**	1.00	
	**13-14**	0.77	0.47-1.26
	**15+**	0.77	0.56-1.07
**Familiarity with addiction**	**No**	1.00	
	**Yes**	0.71	0.46-1.08
**Disease**	**No**	1.00	
	**Yes**	0.68	0.46-1.00
**Brain disease**	**No**	1.00	
	**Yes**	0.82	0.53-1.28
**Biological causes**	**Disagree**	1.00	
	**Neutral/DK**	0.61*	0.41-0.91
	**Agree**	0.71	0.45-1.11
**Social-environmental causes**	**Disagree**	1.00	
	**Neutral/DK**	1.02	0.70-1.48
	**Agree**	1.11	0.74-1.67
**Personal qualities**	**Disagree**	1.00	
	**Neutral/DK**	1.37	0.73-2.56
	**Agree**	2.30***	1.57-3.35

*p < 0.05, **p < 0.01, ***p < 0.001, OR odds ratio, CI confidence interval, DK don’t know.

### Beliefs about stigma, discrimination and dangerousness

Table 6 shows the breakdown of responses for participants’ beliefs about stigma, discrimination and dangerousness. Over 90% of respondents believed that both John and Peter would suffer career damage, that their wives would be likely to leave them and that they would be likely to get into trouble with the law. Stigma scores, as calculated using AMIQ, were significantly higher for persons addicted to alcohol (−4.10, SE = 0.08) than for those addicted to heroin (−6.47, SE = 0.08), indicating that people viewed the heroin addicted person more negatively than the alcohol addicted person (paired *t*-test, p < 0.001).

**Table 6:** **Beliefs relating to stigma, discrimination and dangerousness**

	**Alcohol**			**Heroin**		
	**Affirmative (%)**	**Negative (%)**	**Mean score (AMIQ)**	**Affirmative (%)**	**Negative (%)**	**Mean score (AMIQ)**
John’s/Peter’s career will be damaged by his alcohol/heroin addiction	96.2	3.8	−1.29	98.9	1.1	−1.63
I would be comfortable if John/Peter was my colleague at work	38.5	61.5	−0.31	18.0	82.0	−0.87
I would be comfortable inviting John/Peter to a dinner party	42.9	57.1	−0.22	20.7	79.3	−0.83
How likely do you think it would be for John’s/Peter’s wife to leave him?	92.2	7.8	−1.07	97.3	2.7	−1.43
How likely do you think it would be for John/Peter to get in trouble with the law?	92.3	7.7	−1.21	98.9	1.1	−1.71

Low variation in ratings on the AMIQ items mean that it was not possible to assess associations between demographic factors and stigmatising beliefs about addiction. Answers to questions about the extent to which people felt comfortable interacting with addicted persons in the workplace or at a dinner party showed more variation than answers to questions about the possible negative consequences of addiction. We therefore modelled these separately (Table 7).

**Table 7:** **Beliefs about level of ‘comfort’ with an addicted person**

**I would be comfortable if John/Peter was my work colleague and/or inviting him to a dinner party**		**Alcohol**		**Heroin**	
		**OR**	**95% CI**	**OR**	**95% CI**
**Age**	**18-34**	1.00		1.00	
	**≥35**	0.83	0.51-1.34	1.59	0.91-2.77
**Gender**	**Male**	1.00		1.00	
	**Female**	0.82	0.61-1.10	0.70*	0.51-0.97
**Years of education**	**1-12**	1.00		1.00	
	**13-14**	0.69	0.41-1.16	1.36	0.81-2.28
	**15+**	1.17	0.86-1.60	1.41*	1.00-1.98
**Familiarity with addiction**	**No**	1.00		1.00	
	**Yes**	1.57**	1.16-2.12	1.46	0.98-2.18
**Disease**	**No**	1.00		1.00	
	**Yes**	1.20	0.83-1.73	1.26	0.82-1.93
**Brain disease**	**No**	1.00		1.00	
	**Yes**	1.10	0.77-1.58	0.99	0.63-1.56
**Biological causes**	**Disagree**	1.00		1.00	
	**Neutral/DK**	1.51	1.00-2.27	1.41	0.92-2.18
	**Agree**	1.71**	1.14-2.57	1.34	0.83-2.17
**Social-environmental causes**	**Disagree**	1.00		1.00	
	**Neutral/DK**	1.56*	1.02-2.40	1.78**	1.18-2.69
	**Agree**	1.49	0.99-2.25	1.60*	1.02-2.50
**Personal qualities**	**Disagree**	1.00		1.00	
	**Neutral/DK**	0.69	0.38-1.25	0.74	0.38-1.44
	**Agree**	0.55**	0.39-0.77	0.69	0.47-1.01

*p < 0.05, **p < 0.01, OR odds ratio, CI confidence interval, DK don’t know.

Persons with more familiarity with alcohol addiction were more comfortable with an alcohol addicted person (OR = 1.57, 1.16-2.12). The same was the case for people who agreed that alcohol addiction had biological causes (OR = 1.71, 1.14-2.57). People who were neutral or did not know if alcohol addiction had social-environmental causes were more likely to be comfortable than those who disagreed (OR = 1.56, 1.02-2.40). People who agreed that alcohol addiction was caused by personal qualities were less likely to feel comfortable with an alcohol addicted person than those who disagreed (OR = 0.55, 0.39-0.77).

Different factors predicted the level of comfort people felt with a heroin addicted person. Females were less likely to report feeling comfortable with a heroin addicted person (OR = 0.70, 0.51-0.97) and those with 15 or more years of education were marginally more likely to feel comfortable with someone who had a heroin addiction (OR = 1.41, 1.00-1.98). People who were neutral or did not know whether heroin addiction had social-environmental causes were more likely to feel comfortable with a heroin addicted person than those who disagreed (OR = 1.78, 1.18-2.69). Those who agreed that heroin addiction had social-environmental causes were also more likely to feel comfortable with a heroin addicted person than those who disagreed (OR = 1.60, 1.02-2.50).

## Discussion

Our findings show that public acceptance of the brain disease model of addiction did not reliably predict, either positively or negatively, the attitudinal outcomes we measured. This corroborates our previous qualitative research, which suggested that neuroscientific understandings of addiction were added to, and incorporated into, older ideas about addictions, making it unlikely that new ideas about addiction will produce dramatic shifts in the public’s beliefs about addiction and its treatment [15,16]. It also supports the findings of other studies that the social impacts of neurobiological framings on public understanding are more muted than proponents and critics of these views assume [22,24].

All forms of treatment except medication were endorsed on average by more than 90% of participants. Medication was supported by more than 80% of participants. Participants were highly supportive of all treatment options for both alcohol and heroin addiction, suggesting that the public favours a ‘try anything and everything’ approach.

Predictions that understanding addiction as a brain disease would be associated with greater or lesser support for the use of coerced medical treatment of addicted individuals were not supported by our findings [4,6]. Instead, our findings were consistent with other research findings that views depend very much on whether the drug in question is alcohol or heroin [25].

Support for the belief that addiction is a brain disease did not predict agreement with imprisoning someone addicted to heroin. Support for the belief that addiction is a disease was on the borderline of significance in predicting beliefs about imprisonment for heroin addiction. Support for addiction as a disease or a brain disease was not associated with attitudes towards coerced addiction treatment. Age and education level were more consistent predictors of support for coerced treatment across alcohol and heroin, and age also predicted agreement with imprisonment. This suggests that, overall, life experience (i.e. being 35 years of age or older) and one’s broader knowledge shaped less punitive attitudes towards addicted persons. Women were less likely than men to support coerced treatment of heroin addiction but not alcohol addiction. There was no gender difference in beliefs about imprisonment for heroin use.

Methodological differences between this and previous studies of stigma in addiction limit the ability to make exact quantitative comparisons. However, our pattern of results were consistent with previous studies using the AMIQ [20], where the public viewed persons addicted to heroin significantly more negatively than persons addicted to alcohol. Support for the views that addiction is a disease or a brain disease were not associated with increased feelings of comfort in the company of an addicted person. However, those who endorsed a biological aetiology of alcohol addiction were more likely to be comfortable in the presence of a person addicted to alcohol. This suggests that communicating messages about the biology of alcohol addiction as opposed to a ‘brain disease’ label specifically may reduce stigmatising attitudes to those who suffer from it. Resonating with the concerns of those who advocate a brain disease model, we found that the belief that alcohol addiction was caused by ‘personal qualities’ (such as an ‘addictive personality’, ‘bad character’ and ‘psychological problems’) was more consistently associated with support for coerced treatment of alcohol addiction and decreased comfort with an alcohol dependent person [4].This pattern did not hold for heroin. Our data did not provide evidence that biological explanations, or labels, for addiction would increase stigma [7].

Familiarity with alcohol addiction was associated with increased comfort with someone addicted to alcohol. This pattern did not hold for heroin. This fits with mixed evidence shown in the literature: familiarity can, but does not necessarily, increase comfort levels [26,27]. Being female predicted less comfort with a person addicted to heroin than their male counterparts. There was no effect for alcohol addiction. This equivocal impact of gender on comfort reflects mixed reports found in existing literature [26,28,29].

Having 15 of more years of education was barely significant in predicting comfort with someone addicted to heroin. Educational attainment is not a factor that has received much attention in the literature to date. The lack of a clear pattern in the roles of familiarity, gender and educational level in predicting comfort suggest that more research needs to be done in order to understand these factors and their implications.

Our findings have implications for the communication of information about addiction. The variability in our findings across the drugs alcohol and heroin raises questions about the benefits of formulating messages about the nature of addiction in general. Our findings suggest that we may need to reduce negative views of heroin users and the challenge the public’s tendency to believe that coerced treatment of this group of drug users is justifiable and/or effective [30]. Our findings do not suggest that disseminating messages about the biological basis of addiction in general will achieve this outcome. Recent research by Mannarini and Boffo has shown that addiction is more heavily stigmatised than other mental illnesses [31]. They have hypothesized that this could be explained by beliefs about the *extent* to which addiction is considered voluntary, by comparison to mental illnesses like schizophrenia. They advocate for a more detailed understanding of the factors underpinning ‘social’ versus ‘biological’ causal beliefs [32]. It could also be that it is the interaction of beliefs about different causes of addiction that explains people’s judgements, rather than simple agreement or disagreement to each type of cause (or underlying belief structure) when considered separately. This latter hypothesis would be more consistent with a bidirectional, associative, theory of reasoning than a linear, attributive, theory of reasoning [16]. Examining the effect of the interaction of different beliefs about causes of addiction could be particularly worthwhile, given this sample’s tendency to view addiction as having multiple causes [13].

### Limitations

Methodological differences limit our ability to directly compare our findings on stigma with the previous literature [20]. Our questions varied from the AMIQ in as much as the ‘don’t know/unsure’ responses were placed at the end of the response string in this study rather than in the middle. Second, while the AMIQ was validated for measuring stigma towards mental illness (schizophrenia and depression) and addiction (heroin), we have doubts whether all questions in the AMIQ correctly target stigma of addiction *per se*. Three of the questions are quite similar to the symptoms of substance use disorders specified in the DSM-5 [33]. Thus, responses to these questions could be interpreted as reflecting people’s understandings of the harms of drug and alcohol addiction and/or an awareness of the signs and symptoms of substance use disorders, rather than encompassing morally negative attitudes. Indeed, only the two ‘comfort’ questions in the AMIQ appear to address emotional responses towards addicted persons that may be acted upon to actively stigmatise them by creating social distance.

The primary purpose of this study was to provide a snapshot of the Australian public’s views on the treatment and stigma of addiction, and to do so in a way that yielded results of policy relevance. Latent Class Analysis could be used to achieve a more nuanced understanding of, and address theoretical questions about, the relationships between causal beliefs and stigma [31,34]. In particular, such an approach would allow one to model the effect the substance of abuse has on stigmatising attitudes, directly. Modelling the effect of a specific drug on beliefs about treatments for addiction within a unified model could be a useful approach for health communication, given that limited specificity is practicable when communicating information at a population level.

The overall high level of agreement among respondents in the use of all treatment modalities restricted our ability to undertake statistical analyses to compare beliefs about addiction aetiology and beliefs about treatment. Additionally, we did not ask questions about the public’s belief in the effectiveness of treatments. Further research exploring the public’s beliefs about treatment efficacy is needed.

As we have reported elsewhere, the low response rate and bias in the sample towards older and more educated individuals limits the generalisability of our findings [13]. A low response rate is an increasingly common limitation of surveys conducted using landline telephones because younger people are more likely to use cellular phones [19]. Familiarity with addiction in our sample was based on self-report of one’s own or another’s behaviour and did not quantify the severity. Given the overall low levels of familiarity with heroin addiction, it is possible that a larger sample size could yield different results. Finally, previous research suggests that males and females have divergent views towards addicted males and females [35]. Because our study focussed only on hypothetical addicted males, our findings may not accurately reflect public attitudes towards female drug and alcohol users.

## Conclusion

Our study does not support the more extreme predictions made about either the benefits or the harms of public acceptance of ‘brain disease’ explanations of addiction. The findings suggest a need to communicate messages that destigmatise drug dependent persons, particularly those addicted to heroin. On our data, communication of information about biological causes and understandings of heroin addiction may not yield the large benefits predicted by proponents of this view, although our data suggest a possible benefit from communicating messages about the biology of alcohol addiction.
